# Polyp detection rate in transverse and sigmoid colon significantly increases with longer withdrawal time during screening colonoscopy

**DOI:** 10.1371/journal.pone.0174155

**Published:** 2017-03-22

**Authors:** Kazuhiro Kashiwagi, Nagamu Inoue, Toshifumi Yoshida, Rieko Bessyo, Kazuaki Yoneno, Hiroyuki Imaeda, Haruhiko Ogata, Takanori Kanai, Yoshinori Sugino, Yasushi Iwao

**Affiliations:** 1 Center for Preventive Medicine, Keio University Hospital, Tokyo, Japan; 2 Division of Gastroenterology and Hepatology, Department of Internal Medicine, Keio University School of Medicine, Tokyo, Japan; 3 Department of General Medicine, Saitama Medical University School of Medicine, Saitama, Japan; 4 Center for Diagnostic and Therapeutic Endoscopy, Keio University School of Medicine, Tokyo, Japan; University Hospital Llandough, UNITED KINGDOM

## Abstract

**Background:**

The guidelines for colonoscopy present withdrawal time (WT) and adenoma detection rate (ADR) as the quality indicator. The purpose of this retrospective study is to analyze the predicting factors with polyp detection rate (PDR) as a surrogate for ADR by using comprehensive health checkup data, and assess the correlation between PDR per each colonic segment and WT, and factors influencing WT.

**Methods:**

One thousand and thirty six consecutive health checkup cases from April 2015 to March 2016 were enrolled in this study, and 880 subjects who undertook colonoscopy without polyp removal or biopsy were divided into the two groups (polyp not detected group vs polyp detected group). The two groups were compared by subjects and clinical characteristics with univariate analysis followed by multivariate analysis. Colonoscopies with longer WT (≥ 6 min) and those with shorter WT (< 6 min) were compared by PDR per each colonic segment, and also by subjects and clinical characteristics.

**Results:**

A total of 1009 subjects included two incomplete colonoscopies (CIR, 99.9%) and overall PDR was 35.8%. A multiple logistic regression model demonstrated that age, gender, and WT were significantly related factors for polyp detection (odds ratio, 1.036; 1.771; 1.217). PDR showed a linear increase as WT increased from 3 min to 9 min (r = 0.989, p = 0.000) and PDR with long WT group was higher than that with short WT group per each colonic segment, significantly in transverse (2.3 times, p = 0.004) and sigmoid colon (2.1 times, p = 0.001). Not only bowel preparation quality but also insertion difficulty evaluated by endoscopist were significant factors relating with WT (odds ratio, 3.811; 1.679).

**Conclusion:**

This study suggests that endoscopists should be recommended to take more time up to 9 min of WT to observe transverse and sigmoid colon, especially when they feel no difficulty during scope insertion.

## Introduction

Colonoscopy is the most sensitive and central modality for direct diagnosis of colorectal cancer including cancer screening. However, the rates of missed adenomas by size were reported to be 2.1%, 13%, and 26% for adenomas ≥10 mm, 5–10 mm, and 1–5 mm, respectively, during colonoscopy [1]. Moreover, 6% of invasive colorectal cancers were detected within 3 years of the false-negative index colonoscopy examinations [2]. Thus, evidence- and consensus-based standards for high quality colonoscopy in screening and surveillance programs has been established and recommended in guidelines [3, 4].

Among many quality indicators including bowel preparation, cecal intubation rate (CIR), withdrawal time (WT), sedation and complication rate, adenoma detection rate (ADR) is the only quality indicator that has been demonstrated to be directly associated with interval cancer, which is defined as having been diagnosed between the time of screening colonoscopy and the scheduled time of surveillance colonoscopy [5]. ADR has also been shown to be impacted by various factors including subject age and sex [6], bowel preparation adequacy [7], adequate WT [8, 9] and the individual endoscopist’s performance [10]. A prospective large study involving 2053 subjects of first screening colonoscopies showed a strong direct correlation between WT and all adenoma detection rate (r = 0.90) [8] and a study using multivariate analysis indicated that the endoscopist was more powerful on adenoma detection than the well-established predictions of advancing age or male gender [10]. The Japanese guidelines recommend that diminutive adenomas ≤ 5 mm should be followed up in the absence of colonoscopic findings suggestive of carcinoma [11]. Most of diminutive polyps including adenoma are characterized at real-time using narrow-band imaging, and are not necessarily removed nor diagnosed by pathological examination in the clinical practice [12]. Polyp detection rate (PDR) is readily available from endoscopy reports and has been suggested as a surrogate for ADR [13]. Francis et al. applied a conversion factor to estimate ADR from PDR, and found a strong correlation between estimated ADR and actual ADR [13], but Boroff et al. concluded that PDR and ADR diverged in the distal colon, although they correlated well in the proximal colon [14]. Moreover, recent studies gave renewed importance to proximal lesions, especially small ones. Missed and recurrent adenomas tend to occur in the proximal colon [15] and polyps with features of advanced neoplasia were significantly smaller in the right versus left colon or have a non-polypoid shape, making them likely to be overlooked [16, 17]. More accurate detection of these small and advanced polyps may be required to reduce right-sided colon cancer incidence and mortality. So far there have been no reports stating the correlation between PDR in individual colonic segment and WT. The aim of this retrospective study was to further analyze this correlation by using the data from a large number of subjects in general health checkup. We also tried to assess factors influencing WT.

## Materials and methods

### Study population

The electronic colonoscopy databases were retrospectively searched for 1036 consecutive cases for colorectal cancer screening by colonoscopy between April 2015 and March 2016. Colonoscopies were 260 series variable stiffness instruments (PCF-Q260 AI/AL and PCF-PQ260L (outer diameter of 9.2 mm) with passive-bending and high-force transmission; Olympus Medical System. Tokyo, Japan). Subjects received 2 litters of polyethylene glycol electrolyte solution with a high dose of ascorbic acid (MoviPrep^®^, EA Pharma Co, Tokyo, JPN) [18] and 1 litter of clear fluid according to the manufacture’s instruction. The following data were retrieved from their medical records: demographics (age, gender), medical history, results of colonoscopy including histopathological results, bowel preparation quality, sedation practice, difficulty and pain during colonoscopy insertion evaluated by each of skilled endoscopist. Most of colonoscopies were performed under conscious sedation by giving an intravenous opiate (pethidine hydrochloride) alone, benzodiazepine (midazolam, flunitrazepam) alone, or both. PDR was defined as the percentage of colonoscopies in which at least one polyp was detected per colonoscopy. CIR was confirmed by photo documentation of appendicecal orifice and ileocecal valve. WT was defined as the time taken to withdraw the colonoscopy from the cecal to the anus and recorded as the nearest whole time in unit of one minute. Polyp less than 6 mm was not resected, if it could be diagnosed not to be malignant, and just be followed- up, according to the Japanese guidelines, whereas complete resection of diminutive polyps is recommended in the US to reduce the risk of interval colorectal cancer. The subject with adenomatous polyp ≥ 6 mm was rescheduled to receive a polypectomy by the Japanese National Health Insurance system. Only complete colonoscopies during which no polyps were removed or biopsied were included in analysis to remove the impact of biopsy or therapeutic manoeuvers on the procedure duration, and PDR was calculated per each value of these WTs. Bowel preparation quality was defined as excellent, good, fair, poor, or inadequate, according to a 5 point categorical scale [19]. Insertion difficulty evaluated by the individual endoscopist was classified on four graded scale including easy, slight difficult, difficult, or impossible, according to the number of subject position change, abdominal pressure and /or reinsertion (slight difficult case needed position change and /or abdominal pressure; difficult cases needed position changes and abdominal pressure several times and included reinsertion by PQ scope in exchange for Q scope). This scale was routinely described in our colonoscopy report following each examination to be used as reference to improve subsequent examinations. Pain assessment during insertion by the endoscopist was defined as none, weak, or strong when they needed additional sedation.

The Institutional Review Board of Keio University Hospital approved this retrospective, observational cohort study and the requirement to obtain informed consent was waived (**IRB No. 20160081**).

### Statistical analysis

Statistical difference between the two groups with the presence or absence of polyps was determined by using the Mann-Whitney *U*-test for continuous data and the *χ*^2^ test for categorical data. They included individual subjects and clinical characteristics such as age, gender, past abdominal operation, previous examination, bowel preparation quality, sedation practice, insertion time and WT. The strength of the corresponding associations was estimated by using univariate analysis and multivariate logistic regression analysis, and expressed as odds ratio (OR). The Pearson’s correlation coefficient (r) was used to describe the strength of the association between WT and PDR. All statistical analyses were performed by using SPSS software program (SPSS version 21; SPSS, Inc, Tokyo, JPN). Mean values were expressed with SD. P values < .05 were considered to be statistically significant. A Bonferroni correction was performed where multiple comparisons were undertaken.

## Results

### Subjects and clinical characteristics

**Fig 1:**
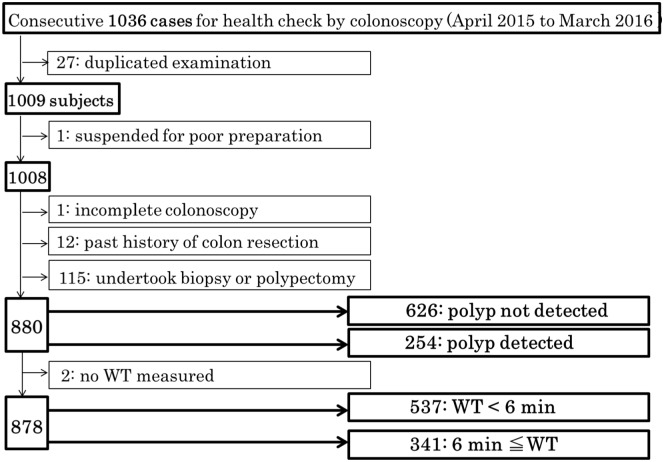
Flow chart of the present study. Flowchart for the selection of study subjects to investigate predicting factors associated with the presence of colorectal polyps, and the association between PDR and WT.

As shown in Fig 1, 27 subjects who undertook colonoscopy two times within one year, were excluded from the 1036 cases. A total of 1009 subjects were analyzed for subjects and clinical characteristics. To calculate the CIR, 1 who had incomplete study due to poor preparation was excluded from the 1009 subjects. Then, 128 subjects were excluded, corresponding to 1 who had incomplete colonoscopy due to a redundant colon, 12 with past history of colon resection, and 115 who undertook biopsy or polypectomy by health insurance treatment from 1008 subjects, in order to investigate the relationship between PDR and WT. As a result of that, 880 subjects were divided into two groups; 626 subjects with polyp not detected and 254 with polyp detected. Moreover, 2 subjects without WT measured were excluded to investigate factors influencing WT. The cecum could be reached in almost all patients (1007/1008; CIR: 99.9%), except one due to a redundant colon, resulting in insertion to mid-ascending colon by PQ scope in exchange for Q scope. Overall PDR and cancer detection rate for 1007 subjects was 35.8% and 0.89% (8 early colon cancer and one carcinoid), respectively. The 14 subjects with adenomatous polyp ≥ 6 mm in size were rescheduled to receive a polypectomy, as the Japanese National Health Insurance system offers coverage for expenses incurred for this procedure.

Subjects and clinical characteristics in 880 cases are summarized in Table 1. The median age was 57.9 ± 11.6 years and 608 subjects were male (69.1%). Nearly 60% subjects had previous colonoscopy examination. No inadequate bowel preparation quality was found and most of bowel prep quality was acceptable (excellent, good, or fair, 97.4%). Only 23% of subjects had the combination of benzodiazepine and opiate, which is widely used as the standard method of gastrointestinal endoscopy for conscious sedation. The average of insertion time was 6.4 ± 4.0 min, whereas insertion difficulty evaluated by endoscopists was as follows (easy, 75.7%; slight difficult or difficult, 24.3%) and these two characteristics did not have strong correlation each other (r = 0.4).

**Table 1:** Main outcome: Subjects and clinical characteristics.

**Subjects**	**n = 880**			
Ages (years)			57.9±11.6 (29–88)	
Gender		male	n = 608	69.1%
		female	n = 272	30.9%
Past abdominal operation		yes	n = 184	20.9%
		no	n = 696	79.1%
		no	n = 372	42.3%
Previous examination		yes	n = 508	57.7%
**Colonoscopy**	**n = 880**			
Bowel preparation quality	n = 806	excellent, good, fair	n = 785	97.4%
		poor	n = 21	2.6%
		inadequate	n = 0	0%
Sedation practice	n = 880	opiate	n = 657	74.7%
		benzodiazepine	n = 3	0.3%
		both	n = 202	23.0%
		none	n = 18	2.0%
Presence of Diverticulosis	n = 880	yes	n = 265	30.1%
		no	n = 615	69.9%
Insertion difficulty	n = 806	easy	n = 610	75.7%
evaluated by endoscopist		slight difficult, difficult	n = 196	24.3%
		impossible	n = 0	0%
Pain evaluated by	n = 800	none	n = 670	83.7%
endoscopist		weak	n = 111	13.9%
		strong	n = 19	2.4%
Insertion time (min)	n = 607		6.4±4.0 (1–40)	
Withdrawal time (min)	n = 878		5.5±2.0 (1–23)	

### Predicting factors associated with the presence of colorectal polyp

Association between patient-related, procedure-related factors and polyp detection are summarized in Table 2. Significantly, more polyps were detected in elderly subjects (OR 1.036, [95%CI 1.022–1.050], p = 0.000), and male dominant (OR 1.771, [95%CI 1.250–2.509], p = 0.001). Of the procedure-related factors, WT was significantly associated with polyp detection (OR 1.217, [95%CI 1.127–1.314], p = 0.000).

**Table 2:** Predicting factors associated with the presence of colorectal polyp.

n = 880	Polyp not detected n = 626		Polyp detected n = 254		Univariable	Multivariable	
					p value	OR (95% C. I.)	p value
Age (years), (SD)	n = 626	56.5(11.9)	n = 254	61.1(10.2)	0.000	**1.036 (1.022, 1.050)**	**0.000**
Male, gender, n (%)	n = 626	411(65.7)	n = 254	197(77.6)	0.001	**1.771 (1.250, 2.509)**	**0.001**
Past abdominal operation (yes), n (%)	n = 626	131(20.9)	n = 254	53(20.9)	0.984		
Previous examination (yes), n (%)	n = 626	353 (56.4)	n = 254	155(61.0)	0.207		
Bowel preparation quality (excellent, good, fair), n (%)	n = 568	550 (96.8)	n = 238	235(98.7)	0.121		
Sedation, (yes), n (%)	n = 626	612 (97.8)	254	250 (98.4)	0.530		
Insertion time (min), (SD)	n = 437	6.5 (4.3)	n = 170	6.1(3.0)	0.149		
Withdrawal time (min), (SD)	n = 624	5.2 (1.9)	n = 254	6.1(2.3)	0.000	**1.217 (1.127, 1.314)**	**0.000**

### Association between PDR and WT

**Fig 2:**
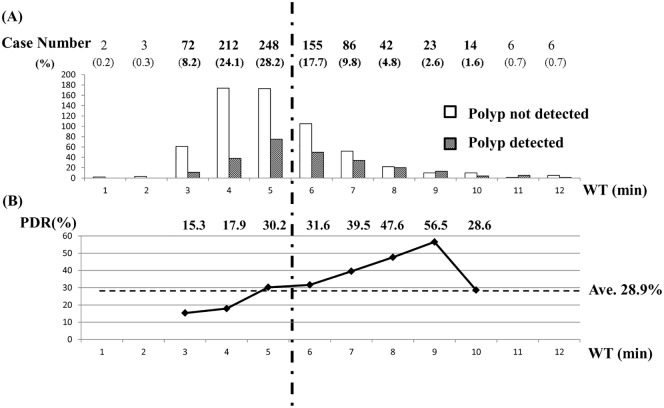
Case number with or without polyp detection, and PDR per each value of WT (min). (A) The left white and right dotted bar graph show case number without polyp detection and with polyp detection, respectively, per each value of WT (minutes). The number of colonoscopy examination per each value of WT, and the percentage of that divided by the total number (n = 878) is indicated above the bar graphs. (B) The polygonal line graph displays PDR per each value of WT. Individual PDR per each value of WT is indicated above the graph. The average PDR for 878 subjects was 28.9%.

In the upper part of Fig 2, left white and right dotted bar show case number of polyp not detected and polyp detected, respectively, per each value of WT (minutes) on the X-axis. The percentage of each case number (polyp detected number plus polyp not detected number) among total case number (n = 878) per each value of WT is given in parentheses. In 61 percent among all examinations, WT was less than 6 min. The lower part of Fig 2 displays that PDR increased almost linearly within the range of WT from 3 min, through 6 min, to 9 min (PDR / WT: 15.3% / 3 min, 31.6% / 6 min, 56.5% / 9 min). The correlation coefficient (r) between PDR and WT from 3 min to 9 min was 0.989 (p = 0.000).

### Association between PDR per each colonic segment and WT, and Factors that influences WT

**Fig 3:**
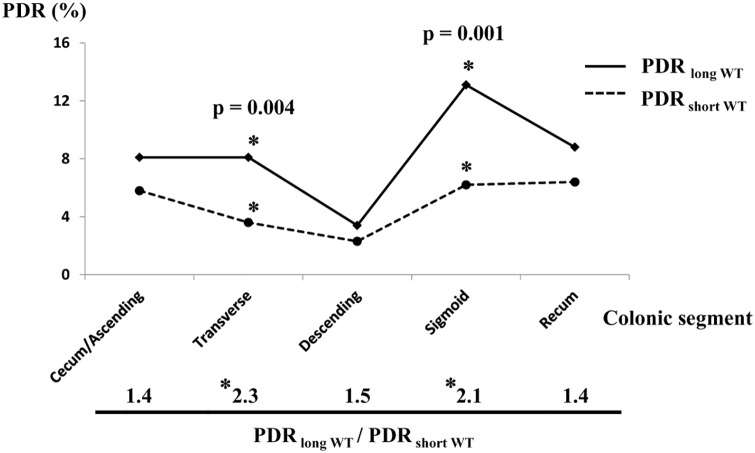
PDR per colonic segment. PDR _long WT_ and PDR _short WT_ stand for PDR of colonoscopies in which WT was ≥ 6 min, and ‹ 6 min, respectively, per each colonic segment. The ratio of PDR _long WT_ divided by PDR _short WT_ is shown below the line graph. Asterisk (*) indicates statistical significance.

Next, PDR per colonic segment was examined between colonoscopies in which WT was ≥ 6 min (PDR _long WT_) and WT < 6 min (PDR _short WT_). AS shown in Fig 3, PDR _long WT_ was higher than PDR _short WT_ in all of five segments, especially in transverse (2.3 times) and sigmoid colon (2.1 times) with a statistical significance (p = 0.004, 0.001). Also, when compared colonoscopies with WT < 6 min to those with WT ≥ 6 min, acceptable bowel prep (excellent, good or fair) and insertion difficulty evaluated by endoscopist (easy) were significant factors relating with WT < 6 min (OR 3.811, [95% CI 1.439–10.096], p = 0.007; OR 1.679, [95% CI 1.204–2.341], p = 0.002). However, there was no correlation between WT and insertion time by univariate analysis (p = 0.880) (Table 3).

**Table 3:** Factors that influence WT.

n = 878	WT < 6 min (4.3±0.7 min) n = 537		WT ≥ 6 min (7.3 ±2.1 min) n = 341		Univariable	Multivariable	
					p value	OR (95% C. I.)	p value
Age (years), (SD)	n = 537	57.5(1.7)	n = 341	58.5(11.4)	0.218		
Male, gender, n (%)	n = 537	364(67.8)	n = 341	243(71.3)	0.277		
Past abdominal operation (yes), n (%)	n = 537	119(22.2)	n = 341	63(18.5)	0.189		
Previous examination (yes), n (%)	n = 537	303(56.4)	n = 341	203(59.5)	0.364		
Bowel preparation quality (excellent, good, fair), n (%)	n = 503	497(98.8)	n = 302	287(95.0)	0.001	**3.811 (1.439, 10.096)**	**0.007**
Presence of Diverticulosis (yes), n (%)	n = 537	159(29.6)	n = 341	106(31.1)	0.642		
Insertion difficulty evaluated by endoscopist (easy), n (%)	n = 503	402(79.9)	n = 301	207(68.8)	0.000	**1.679 (1.204, 2.341)**	**0.002**
Pain evaluated by endoscopist (none), n (%)	n = 501	421(84.0)	n = 298	248(83.2)	0.764		
Insertion time (mn), (SD)	n = 336	6.4(4.1)	n = 271	6.3(3.7)	0.880		

WT: Withdrawal Time

There was neither sedation-related complication, nor procedure-related complication including bleeding and perforation in this study.

## Discussion

More recently, WT as well as ADR have been presented to be directly associated with the outcome measure, interval colorectal cancer [5, 20]. The U.S. Multi-Society Task Force on Colorectal Cancer recommends 6 to 10 min as the minimal amount of time needed for adequate inspection during the withdrawal phase; minimum ADR of 25% (30% of men and 20% of women aged 50 or older) in persons undergoing first-time examinations [21, 22]. However, there are conflicting reports on the associations between WT and the number of detected polyps [23, 24]. Moreover, mean WT longer than 6 min may not be associated with increased detection of advanced adenomas [25]. Because an adequate withdrawal phase is thought to be an essential feature of quality colonoscopy, there has been interest in withdrawal technique that can improve ADR [26]. Withdrawal technique such as repositioning the patient, adequate distension, suction, leaning, meticulous mucosal re-inspection, careful examination of the proximal aspect of folds, and rectal retroversion, may be more important than WT [27] and it may not be necessarily associated with WT. Furthermore, some endoscopists may perform a longer, more detailed examination on insertion and require less time on withdrawal.

The very high CIR (99.9%) and high percentage of acceptable bowel preparation quality (98.4%) in our study is evidence of the high technical quality and compare favorably with proposed standards and targets by the Bowel Cancer Screening Programme [28]. But, the average of WT in our institution was 5.5 min, contrary to the guidelines [3, 4]. As we didn’t always get pathological evidence for all of the diminutive polyps [12], we tried to analyze the related factors with the presence of polyp and the possibility of whether colonoscopies of WT ≥ 6 min could contribute to more polyp detection, and found three results as follows. First, we demonstrated that PDR increased almost linearly within the range of WT from 3 min to 9 min with very strong correlation coefficient, which is alike with previous study results for ADR [8, 9, 25]. Additionally, Lee et al reported that the increase in ADR is minimal beyond 10 min [25] and this limitation seems to be alike with our study suggesting that the increase in PDR may be minimum beyond 9 min. Second, when colonoscopies were grouped into the two levels of WT (WT < 6 min, or 6 min ≤ WT), the long WT group contributed to higher PDR than the short WT group, especially for polyp detection in transverse and sigmoid colon with a statistical significance. These two colon segments are not fixed unlike the other segments, and have steer angulation including sigmoid-descending junction and hepatic flexure, resulting in the increased rate of missed polyps, specifically small ones, in short period of observation. The association between PDR and each colonic segment in our study is very similar to that shown by the former report [14]; PDRs are higher in the sigmoid and rectum. This distribution of polyps through the colorectum may be almost constant beyond races for subjects with average-risk of colorectal cancer. Third, not only acceptable bowel preparation but also insertion difficulty evaluated by endoscopist was significant factors relating with WT. This latter finding could reflect the fact that it takes a longer time to observe the entire colon especially when transverse and/or sigmoid colon are longer and more tortuous than usual, have more sharp angulation, and are easy to make excessive looping, therefore the endoscopist might feel difficulty and get stressed during scope insertion into proximal portion for the same reasons. Surprisingly, there was a stronger correlation between WT and reported difficulty of insertion, rather than between insertion time and difficulty of insertion. It is partially because we couldn’t count insertion time in many cases (880–607 = 281 missing cases) including 19 cases which were classified as “difficult”, as a scope exchange was needed. Additional univariate analysis found no direct association between the presence of colorectal polyp and insertion difficulty (p = 0.733). Therefore, a prospective study is needed to investigate the association between PDR and WT per each colonic segment, specifically in transverse and sigmoid colon, and to verify the validity of this scale as a predictor of WT, after having the same type of sedation used.

Our study has some limitations including its inherent retrospective design and incomplete characterization of histology of polyps. To present the association between ADR and WT, we carefully reexamined the photographs taken by narrow-band imaging for all of the detected polyps in 254 subjects according to the narrow-band imaging international colorectal endoscopic (NICE) classification [29, 30]. As a result, nearly half of diminutive polyps were judged as being adenomatous by optical diagnosis, of which the result is similar to the recent report showing high accuracy of optical diagnosis for diminutive polyps and 53% of diminutive polyps diagnosed as being adenomatous [31]. Also, colonoscopies with long WT contributed to higher ADR in all of each colonic segment, but there was no statistical significance, compared with colonoscopies with short WT (S1 Fig). Our results also suggest that PDR overestimated ADR by in the distal colon, essentially not a realistic predictor, as shown in the comparison of Fig 3. and S1 Fig. A disadvantage in using PDR as a surrogate for ADR is the risk that endoscopist may perform more biopsies of “lesions”, not necessarily increasing adenoma detection, so-called the “gaming”. It should be emphasized that not only polyp detection but also optical diagnosis for differentiating adenoma from hyperplastic polyp is significant.

Second, nearly 90% of polyps detected in 254 subjects in our study were diminutive. Polyps with advanced pathology were 5-folds more likely to be < 6 mm in the right vs left colon [16] and these diminutive advanced adenomas might be one of the main cause of interval cancer. It remains to be undefined even after optical diagnosis whether diminutive polyps found in our study may have advanced pathology or not. It’s also beyond the scope of this study whether our finding can be applied to larger polyps, although some reports demonstrated that longer WT had weaken or no association with the detection for larger polyps (≥ 6 mm) or advanced adenomas [9, 25]. Third, up to 97% of colonoscopies were withdrawn within 10 minutes in this study and we couldn’t examine the effect of WT which is longer than 10 minutes for PDR.

In conclusion, we recommend taking more time for observation of transverse and sigmoid colon, especially when endoscopist feel no insertion difficulty, resulting in approximately up to 9 minutes of WT in order to maximize PDR.

## Supporting information

S1 Fig ADR per colonic segment. (TIF)
