# Supplementary figures and images for: Polyp detection rate in transverse and sigmoid colon significantly increases with longer withdrawal time during screening colonoscopy
Source: PLoS One. 2017 Mar 22;12(3):e0174155. doi: 10.1371/journal.pone.0174155 (PMC5362195; doi:10.1371/journal.pone.0174155)

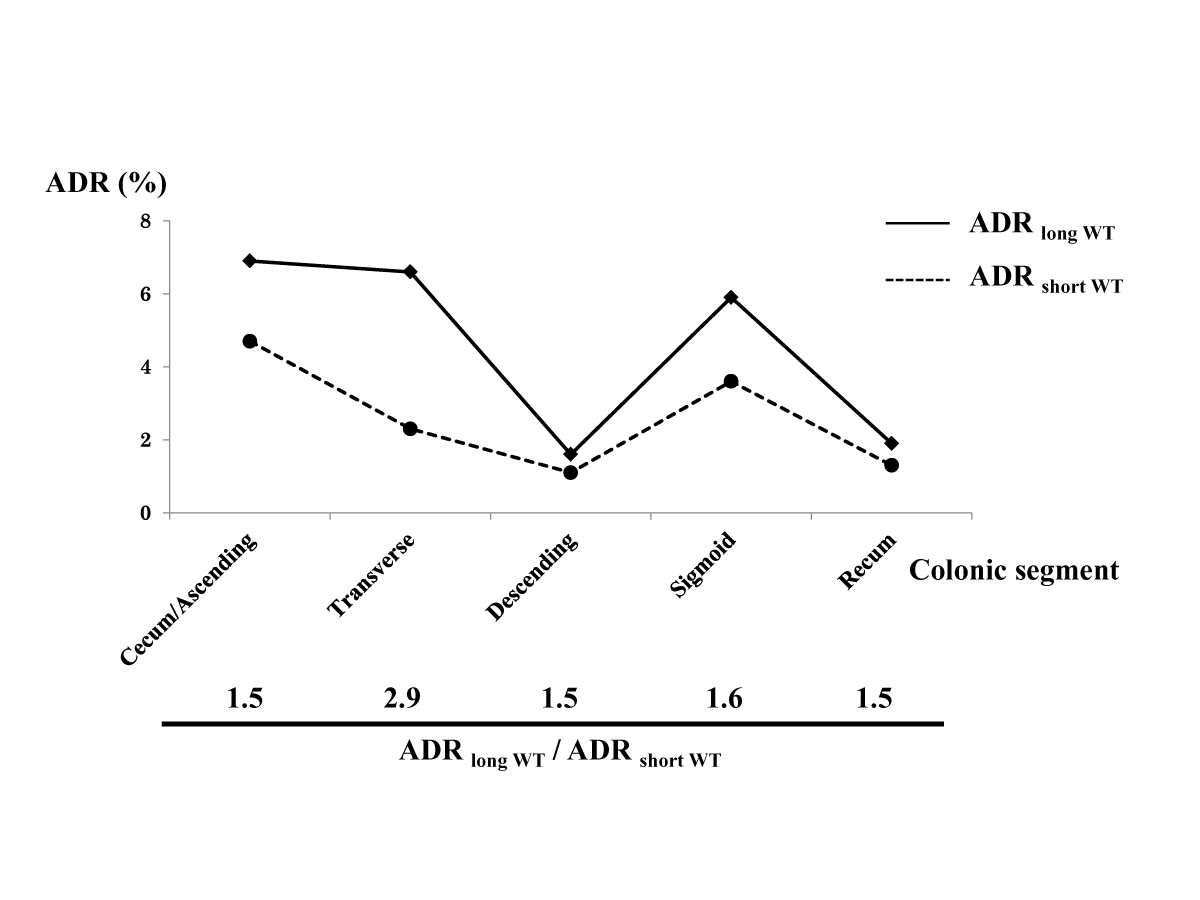

Supplement: S1 Fig — (TIF) [file pone.0174155.s001.tif]
